# Variation of chemical compounds in wild Heliconiini reveals ecological factors involved in the evolution of chemical defenses in mimetic butterflies

**DOI:** 10.1002/ece3.6044

**Published:** 2020-02-13

**Authors:** Ombeline Sculfort, Erika C. P. de Castro, Krzysztof M. Kozak, Søren Bak, Marianne Elias, Bastien Nay, Violaine Llaurens

**Affiliations:** ^1^ Institut de Systématique, Evolution, Biodiversité (ISYEB) Muséum National d'Histoire Naturelle CNRS Sorbonne‐Université EPHE Université des Antilles Paris France; ^2^ Unité Molécules de Communication et Adaptations des Micro‐organismes (MCAM) Muséum National d'Histoire Naturelle CNRS Paris France; ^3^ Department of Zoology Cambridge University Cambridge UK; ^4^ Smithsonian Tropical Research Institute Panamá Panamá; ^5^ Department of Plant and Environmental Sciences University of Copenhagen Frederiksberg Denmark; ^6^ Laboratoire de Synthèse Organique Ecole Polytechnique CNRS ENSTA Institut Polytechnique de Paris Palaiseau Cedex France

**Keywords:** aposematism, cyanogenic glucosides, *Heliconius*, LC‐MS/MS, Müllerian mimicry, phylogenetic signal

## Abstract

Evolutionary convergence of color pattern in mimetic species is tightly linked with the evolution of chemical defenses. Yet, the evolutionary forces involved in natural variations of chemical defenses in aposematic species are still understudied. Herein, we focus on the evolution of chemical defenses in the butterfly tribe Heliconiini. These neotropical butterflies contain large concentrations of cyanogenic glucosides, cyanide‐releasing compounds acting as predator deterrent. These compounds are either de novo synthesized or sequestered from their *Passiflora* host plant, so that their concentrations may depend on host plant specialization and host plant availability. We sampled 375 wild Heliconiini butterflies across Central and South America, covering 43% species of this clade, and quantify individual variations in the different CGs using liquid chromatography coupled with tandem mass spectrometry. We detected new compounds and important variations in chemical defenses both within and among species. Based on the most recent and well‐studied phylogeny of Heliconiini, we show that ecological factors such as mimetic interactions and host plant specialization have a significant association with chemical profiles, but these effects are largely explained by phylogenetic relationships. Our results therefore suggest that shared ancestries largely contribute to chemical defense variation, pointing out at the interaction between historical and ecological factors in the evolution of Müllerian mimicry.

## Graphical abstract

Within Heliconiini butterflies, we show that ecological factors such as mimetic interactions and host plant specialization have a significant effect on chemical profiles, but these effects are largely explained by phylogenetic relationships. Our results suggest that shared ancestries largely contribute to chemical defence variation, pointing out at the interaction between historical and ecological factors in the evolution of Müllerian mimicry.

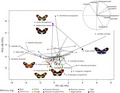

## 1 INTRODUCTION

The evolution of complex phenotypes combining different traits subject to natural selection raises the question of the mechanisms underlying adaptation involving multiple traits. In aposematic species for instance, the defensive traits such as toxicity, and the warning coloration may evolve asynchronously and can be submitted to contrasted selective pressures. While the evolution of color patterns and the selective mechanisms involved have received considerable attention (Le Poul et al., 2014; Sherratt, 2008), the evolutionary origin of chemical defense variations is still understudied. The effect of chemical defenses on predator avoidance is critical for prey survival (Ihalainen, Lindström, & Mappes, 2007) and therefore central in the evolution of warning colorations (Blount, Speed, Ruxton, & Stephens, 2009; Speed & Ruxton, 2007). By sampling aposematic prey, predators learn to associate deterrent effect with a given warning color pattern and subsequently avoid any resembling prey item (Alcock, 1970a, 1970b; Goodale & Sneddon, 1977). The immediate and long‐term effect of defensive compounds thus determines the protection gained from aposematism (Skelhorn & Rowe, 2005), and therefore the evolution of color patterns.

Evolutionary convergence in aposematic signal among co‐occurring defended prey species is frequently observed among sympatric aposematic species, because sharing a color pattern decreases individual predation risk (Müller, 1879). This results in so‐called mimicry rings, composed of multiple species sharing a similar warning color pattern. Both the defensive compounds and the abundance of individuals sharing a given warning color pattern determine the predation risk associated with this coloration (Sherratt, 2008). Substantial quantitative variation in chemical defenses is observed between mimetic species, as demonstrated for instance in poison frogs (Santos & Cannatella, 2011), marine gastropods opisthobranchs (Cortesi & Cheney, 2010), or insects (Arias et al., 2016; Bezzerides, McGraw, Parker, & Husseini, 2007; Castro, Zagrobelny, et al., 2019). Less defended individuals may act as parasites on better defended individuals by limiting predator avoidance (Rowland, Mappes, Ruxton, & Speed, 2010; Speed, 1993)*.* The evolution of chemical defenses in mimetic species is thus likely to be influenced by the local abundance of the mimicry ring they belong too, as well as variations in toxin levels across individuals composing the ring.

Nevertheless, other local ecological factors may influence the evolution of chemical defenses in mimetic species. In butterflies for instance, deterrent compounds, as well as precursors for their synthesis, can be acquired by caterpillars during feeding on specific host plants (Jones, Petschenka, Flacht, & Agrawal, 2019; Nishida, 2002). Chemical defenses may thus vary among species depending on their diet (Engler & Gilbert, 2007). For instance, monarch butterflies (*Danaus plexippus*) sequester cardenolides from milkweeds during the larval stage and are thus unpalatable to birds (Brower, McEvoy, Williamson, & Flannery, 1972). Adaptation to host plants is thus a key evolutionary factor in the origin and evolution of chemical defenses in aposematic butterflies. Nevertheless, because of the strength of predation on adult butterflies, the evolution of chemical defenses in mimetic butterflies can result from complex interactions between host plant adaptation and predation pressure. A recent survey of natural populations of two comimetic butterfly species, the viceroy (*Limenitis archippus*) and queen (*Danaus gilippus*), demonstrated that the average concentration of chemical defenses increases in the viceroy populations where the defended queen species is absent (Prudic, Timmermann, Papaj, Ritland, & Oliver, 2019). This effect is independent from variation in defensive compounds concentrations in the host plants (Prudic et al., 2019), highlighting that the abundance of comimics may modulate selection exerted on chemical defenses in mimetic species.

**Figure 1:**
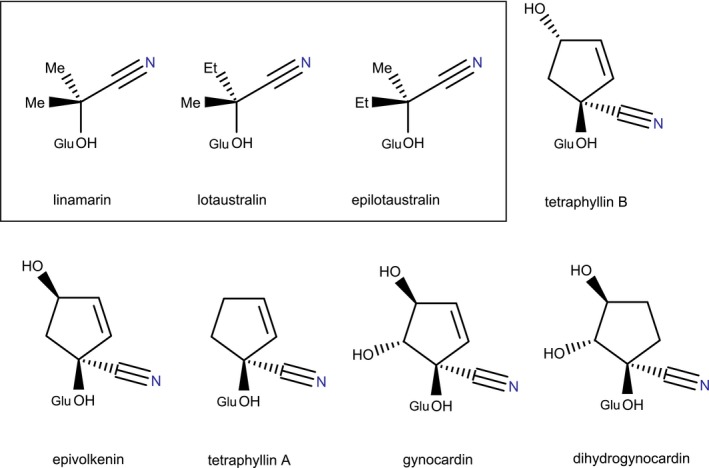
CGs identified in Heliconiini. Framed molecules are aliphatic CGs synthesized by Heliconiini, followed by cyclopentenoid CGs sequestered from *Passiflora* plants. Glucose group is symbolized by “Glu.” For the first time in Heliconiini, we report epilotaustralin and a stereoisomer of tetraphyllin A (putatively the deidacline, which is not represented here because it was not firmly identified during this study)

Here, we aim to disentangle the mechanisms involved in the evolution of chemical defenses, from neutral divergence to selective pressure of predation and host plant adaptation. We focus on the butterflies belonging to the neotropical tribe Heliconiini (Nymphalidae: Heliconiinae), where color pattern evolution and mimetic interactions have been extensively documented (Joron & Iwasa, 2005; Joron & Mallet, 1998; Merrill et al., 2015). Subspecies of Heliconiini are defined based on variation in color pattern between geographic locations, observed within species (Braby, Eastwood, & Murray, 2012). Heliconiini butterflies contain a wide diversity of defensive compounds, especially aliphatic or cyclopentenoid CGs (CGs) (Figure 1) (Castro, Zagrobelny, et al., 2019; Engler, Spencer, & Gilbert, 2000). CGs are supposed to have a bitter and repulsive taste (Nahrstedt & Davis, 1985). Additionally, CGs release toxic cyanide and chemical by‐products for birds when put in contact with specific degrading enzymes (Cardoso, 2019; Conn, 1980). CGs and enzymes or stored in different cell or tissue compartment and are mixed upon tissue disruption under a predator's attack, so that Heliconiini butterflies often survive an attack after being tasted (e.g., by lizard (Boyden, 1976) or avian predators (Boyden, 1976; Chai, 1996; Pinheiro & Campos, 2019)). Therefore, the bitter taste provided by CG and toxic metabolites may act as a chemical defense because of immediate deterrent effect on predator.

Heliconiini caterpillars feed on *Passiflora* plants (Engler & Gilbert, 2007; Jiggins, 2016; Turner, 1967), with substantial behavioral variation between species in female egg‐laying preferences and in larval survival on different *Passiflora* species (Benson, Brown, & Gilbert, 1975; Brown, 1981). Around 30 different CGs have been identified in *Passiflora* (Castro, Zagrobelny, et al., 2019; Spencer & Seigler, 1987). Larvae of most Heliconiini species synthesize CGs de novo (Wray, Davis, & Nahrstedt, 1983), but many sequester CGs from the host plants (Engler et al., 2000). Both synthesis and sequestration of CGs are only observed in Zygaenidae (burnet moths) and Heliconiini, two clades where aposematic color patterns have evolved (Zagrobelny, Castro, Møller, & Bak, 2018). So far, Heliconiini have been reported to sequester five cyclopentenoid CGs from *Passiflora*; the diastereoisomers tetraphyllin B and epivolkenin, tetraphyllin A, gynocardin, and dihydrogynocardin (Figure 1) (Castro, Zagrobelny, et al., 2019; Engler et al., 2000). Heliconiini butterflies can synthesize aliphatic CGs, linamarin, and lotaustralin (Figure 1) from the amino acids valine and isoleucine, respectively (Nahrstedt & Davis, 1985). Identifying the different CGs may thus allow tracking down their metabolic origins, although aliphatic linamarin and lotaustralin can also be uptaken by caterpillars, as recently demonstrated in *Heliconius melpomene* (Castro, Demirtas, et al., 2019). The balance between sequestration from host plants and de novo synthesis of CGs in different species may be linked to host plant specialization. CG sequestration might be more important than synthesis in specialist species, as for instance in the specialist species *Heliconius sara* and *H. sapho* containing drastically diminished CG concentrations when reared on *Passiflora* species other than their specific host plants (Engler & Gilbert, 2007). Evolution of chemical defenses in the Heliconiini clade can thus be influenced by the adaptation to host plants.

The substantial geographic variation in color patterns and host plants observed in the Heliconiini clade (Jiggins, 2016) provides a relevant opportunity to investigate the effect of selection pressure on the evolution of chemical defenses in mimetic species. Based on the well‐studied phylogeny of Heliconiini (Kozak et al., 2015), we thus explored how phylogenetic history, mimetic interactions, and host plant use can drive the evolution of chemical defense in wild butterflies. We sampled butterflies throughout Heliconiini distribution, from Central to South America, in order (a) to maximize the diversity of species of the Heliconiini clade (we cover almost half of the tribe diversity), and (b) to assess variation in chemical defenses of individuals facing natural variations in host plant availability, mimetic community abundance, and predator communities. Using liquid chromatography coupled to mass spectrometry (LC‐MS/MS), we investigate both quantitative and qualitative variation across individuals and then use comparative methods to disentangle phylogenetic and ecological factors influencing the evolution of chemical defenses in Heliconiini.

## 2 MATERIALS AND METHODS

### 2.1 Butterfly collection

We sampled butterflies throughout Heliconiini distribution to collect the maximum number of species. Wild butterflies were caught from 2016 to 2018 across Peru (*n* = 286), Panama (*n* = 45), Ecuador (*n* = 24), and Brazil (*n* = 20), using a hand net. We used 375 individuals from 33 species, covering 43% of the Heliconiini tribe (Appendix 1), and 55 subspecies (Table 1). Individuals were killed by freezing on the day of capture (approximately −18°C). Wings were cut at their attachment point to the body and preserved dried in an envelope and placed in a silica gel containing box to absorb humidity. In order to preserve the integrity of CG molecules, bodies were conserved in a plastic vial containing 100% methanol and kept in freezer (approximately −18°C).

**Table 1:** Subspecies are divided in nine mimicry rings

Mimicry ring	Subspecies
Blue 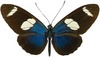	*Heliconius congener congener*
	*Heliconius doris doris*
	*Heliconius doris viridis *(blue morph)
	*Heliconius sara magdalena*
	*Heliconius sara sara*
	*Heliconius wallacei flavescens*
Dennis ray 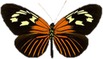	*Eueides tales calathus*
	*Heliconius aoede cupidineus*
	*Heliconius burneyi jamesi*
	*Heliconius demeter joroni*
	*Heliconius erato emma*
	*Heliconius eratosignis ucayalensis*
	*Heliconius melpomene aglaope*
	*Heliconius timareta timareta*
	*Heliconius xanthocles melior*
	*Heliconius xanthocles zamora*
Green 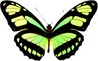	*Philaethria diatonica*
	*Philaethria dido dido*
	*Philaethria dido panamensis*
Orange 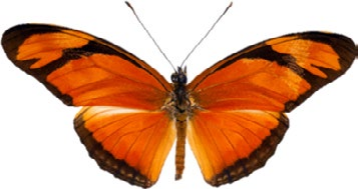	*Agraulis vanillae luciana*
	*Agraulis vanillae vanillae*
	*Dione juno huascuma*
	*Dione juno miraculosa*
	*Dryadula phaetusa*
	*Dryas iulia moderata*
	*Eueides aliphera aliphera*
	*Eueides lybia lybia*
Postman Panama 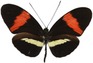	*Heliconius erato demophoon*
	*Heliconius melpomene rosina*
Postman Ecuador/Peru 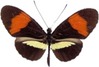	*Heliconius erato favorinus*
	*Heliconius melpomene amaryllis X aglaope*
	*Heliconius telesiphe sotericus*
	*Heliconius timareta thelxinoe*
Postman reverse 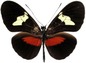	*Heliconius himera* *Heliconius timareta timareta*
Rayed yellow 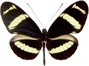	*Heliconius hewitsoni*
	*Heliconius pachinus*
Tiger 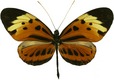	*Eueides isabella dissoluta*
	*Eueides isabella hippolinus*
	*Eueides lampeto acacetes*
	*Heliconius ethilla aerotome*
	*Heliconius hecale felix*
	*Heliconius numata arcuella*
	*Heliconius numata lyrcaeus*
	*Heliconius numata tarapotensis*
	*Heliconius numata zobryssi*
	*Heliconius pardalinus butleri*
	*Heliconius pardalinus sergestus*
Other	*Heliconius melpomene amaryllis X aglaope*
	*Eueides isabella eva*
	*Heliconius charithonia vazquezae*
	*Heliconius doris viridis *(red morph)
	*Heliconius eleuchia primularis*
	*Heliconius erato cyrbia*
	*Heliconius hecale melicerta*
	*Heliconius hecale zuleika*
	*Heliconius numata bicoloratus*

Note

Geographically isolated, phenotypically unique, and hybrid individuals were assigned to “Other.” Subspecies belonging to the same mimicry ring share a given colour pattern within the same locality. Mimicry rings and subspecies within are listed in alphabetical order.

### 2.2 Cyanogenic glucoside extraction in methanol

For each butterfly specimen, the butterfly body and the methanol medium were transferred in a glass tube. Methanol was evaporated at room temperature until the tissue was fully dried using Savant Automatic Environmental SpeedVac System AES1010 with VaporNet. For each specimen, body and wings were weighed before being crushed together into a fine powder in a glass mortar and pestle using liquid nitrogen. Two mL of 100% methanol was added to the powder before stirring for 1 hr at room temperature. Extracts were centrifugated for 20 min at 4,500 *g*, filtered using 7 mm diameter glass pipettes and cotton, filtered again with a MultiScreen 0.45 µm hydrophilic, low protein binding plate, and centrifuged five minutes at 10,000 *g*. Raw filtrates were diluted 50 times in milliQ water, vortexed, and stored in fridge until liquid chromatography and tandem mass spectrometry (LC‐MS/MS) injections.

### 2.3 Liquid chromatography and tandem mass spectrometry

The protocol used in this study has been previously optimized to identify and quantify CGs in butterfly methanol filtrates (Briolat, Zagrobelny, Olsen, Blount, & Stevens, 2019; Castro, Zagrobelny, et al., 2019). Analytical LC‐MS/MS was performed using an Agilent 1,100 Series LC (Agilent Technologies, Germany) coupled to a High Capacity Trap‐Ultra ion trap mass spectrometer (Bruker Daltonics, Germany). Chromatographic separation was carried out on a Zorbax SB‐C18 column (Agilent; 1.8 μM, 2.1 × 50 mm). Mobile phase A was composed by deionized water containing 0.1% (v/v) formic acid. Mobile phase B was acetonitrile supplemented with 50 μM NaCl and 0.1% (v/v) formic acid. The gradient was as follows: 0–0.5 min, isocratic 2% B; 0.5–7.5 min, linear gradient 2%–40% B; 7.5–8.5 min, linear gradient 40%–90% B; 8.5–11.5 isocratic 90% B; 11.6–17 min, isocratic 2% B. Flow rate was set to 0.2 ml/min and increased to 0.3 ml/min between 11.2 and 13.5 min. During the liquid chromatography step, initially neutral CGs were associated with Na^+^ cations and analyzed with mass spectrometer in the positive electrospray mode. The oven temperature was fixed at 35°C.

In addition to the 375 butterfly samples, we ran blank control sample and a reference sample. Blank was methanol gone through the whole protocol extraction, and the reference sample was a mix of every butterfly filtrates. CGs were identified by comparison to standard solutions (aliphatic were chemically synthesized at PLEN, Møller, Olsen, & Motawia, 2016, cyclopentenoid were donated by Lawrence Gilbert and Helene Engler, Engler et al., 2000). We made three calibration curves based on three commercial standards: linamarin, lotaustralin/epilotaustralin, and amygdalin (commercial, Sigma Aldrich), from 0.1 to 20 ng/µl each. Blanks, standards, calibration curve, and reference sample were run first. The reference sample was injected every ten butterfly samples.

### 2.4 Chemical data analyses

Mass spectra were analyzed using the software Bruker Compass DataAnalysis 4.3 (x64). We targeted sodium adducts [M + Na^+^] of linamarin [retention time (RT) 2.4 min at *m/z* 270], lotaustralin [RT 5.4 min at m/z 284], epilotaustralin [RT 5.5 min at m/z 284], tetraphyllin B [RT 1.3 min at m/z 310], epivolkenin [RT 2.3 min at *m/z* 310], tetraphyllin A [RT 4.9 min at *m/z* 294], gynocardin [RT 1.4 min at *m/z* 326], dihydrogynocardin [RT 1.4 min at *m/z* 328], and amygdalin [RT 6.4 min at *m/z* 480] (Briolat et al., 2019; Castro, Zagrobelny, et al., 2019). For every targeted CG compound, the total concentration was estimated based on the extracted ion chromatogram (EIC) peak areas, and on a regression calculated from the standard curve (in ng of CG/mL of butterfly extract). We reported the concentration of each CG in every butterfly in µg of CG/mg of dried butterfly weight.

### 2.5 Statistical and comparative analyses

For each individual, we obtained the concentration of each of the nine studied CGs, referred to as the chemical profile. By adding these nine CG concentrations, we computed the total CG concentration per individual, as an estimation of the amount of chemical defenses per individual. All statistics were conducted in R 3.4.4 (R: The R Project for Statistical Computing, 2019a) and RStudio 1.1.463 (RStudio, 2019b). Plots were created with *ggplot2* 3.0.0 package (Wickham et al., 2019).

#### 2.5.1 Qualitative and quantitative variation in CGs

We used MANOVA (Multivariate ANalysis Of Variance) to test whether the (multivariate) CG profiles were different between groups (genera, species, and subspecies), and we reported the name of the test, Pillai's trace, degree of freedom, and associated *p*‐value. We used the Pillai's test because of its robustness regarding heterogeneities in variance–covariance.

We used ANOVA (ANalysis Of Variance) to test whether the concentration of a specific CG was different between groups. We presented statistical result of ANOVA as follow: name of the test, *F* value (variance of the group means/ mean of the within group variances), degree of freedom, and associated *p*‐value. In case of a significant ANOVA (*p*‐value < .050), post hoc test Tukey Honest Significant Differences (Tukey's HSD) was done to determine which group was significantly different from the others. Statistical tests were run with R package *stats* 3.4.2. Heatmap of CG occurrence and concentration was plotted using R packages *ape* 5.1 and *ggtree* 1.10.5 (Paradis, 2011; Yu, Smith, Zhu, Guan, & Lam, 2017).

#### 2.5.2 Evolution of cyanogenic glucoside profiles in Heliconiini

We calculated the phylogenetic signal of CG profile, *that is*, the extent to which trait values are explained by the phylogeny, or how much closely related species resemble one another in terms of CG profile (Blomberg, Garland, & Ives, 2003). We computed the *K*_mult_ statistic, a multivariate extension of Blomberg's *K* test for univariate phylogenetic signal (Adams, 2014; Blomberg et al., 2003). A low phylogenetic signal (*K*_mult_ close to 0) indicates a low influence of the phylogenetic relationships on the tested trait, whereas high value (*K*_mult _close to 1) suggests that the trait evolution along the phylogeny is close to Brownian motion. The multivariate phylogenetic signal of quantitative CG variation across species was evaluated using *K*_mult_ in the *geomorph* 3.0.7 R package. We calculated the phylogenetic signal in the whole Heliconiini tribe, in the largest genus of the radiation: *Heliconius* and more specifically in ancient nodes (pupal‐mating and nonpupal‐mating clades). In *Heliconius*, phenotypic races of the same species often belong to different mimicry rings. Therefore, we estimated the phylogenetic signal using mean CG concentrations separately at the taxonomic level of species (*n* = 33) and subspecies (*n* = 55). We adapted the Heliconiini phylogenetic tree (Kozak et al., 2015) by pruning species not represented in our sample set. In many cases, several subspecies were sampled (for example: *H. hecale felix, H. hecale melicerta, and H. hecale zuleika*). For the subspecies‐level analysis, we extended the original phylogeny to include relevant subspecies as follows: the terminal branch length was set equal to the decimal of the previous branch, and the common branch equal to the integer part. All subspecies had same total branch length. In the case of more than two subspecies, the topology was arbitrary resolved.

#### 2.5.3 Phylochemospace

We applied the concept of phylomorphospace, describing morphological variation across species in correlation with their phylogenetic relationships (Sidlauskas, 2008). We built a “phylochemospace” describing variation in concentration of multiple compounds with a principal component analysis (PCA), superimposing the phylogenetic relationships among subspecies. The resulting PCA visualizes the variation in CGs actually occurring in the 55 subspecies. Packages *FactoMineR* 1.41 (Lê, Josse, & Husson, 2008), *missMDA* 1.14 (Josse & Husson, 2016), and *phytools* 0.6‐44 (Revell, 2012) were used.

#### 2.5.4 Variation among comimetic subspecies and host plant specialization

We tested for differences between groups: mimicry ring, geographical range, and host plant specialization. We used MANOVA and ANOVA to assess differences in CG profile and specific CG concentrations, respectively, both at species (*n* = 33) and subspecies (*n* = 55) level. We applied Bonferroni correction as we performed several tests on the same dataset. We used *stats* 3.4.2 for MANOVA and *RVAideMemoire* 0.9‐72 package (Hervé, 2019) for associated post hoc test. ANOVA, associated post hoc test, and Bonferroni correction were computed with *stats* 3.4.2 package as well.

To assess whether the observed statistically significant differences were due to shared ancestry, we computed phylogenetic MANOVA and ANOVA, using *geiger* 2.0.6 (Harmon, Weir, Brock, Glor, & Challenger, 2008) and *phytools* 0.6‐44 packages (Revell, 2012), respectively. Phylogenetic MANOVA was performed using the modified tree and mean CG concentrations per subspecies (as these phylogenetical tests do not handle multiple values for one subspecies, we used mean concentrations).

We investigated variation in total CG concentration, putatively synthesized CG concentration, and putatively sequestered CG concentration between generalist and specialist subspecies. When considering the entire range of a given species across Central and South America, it turns out it can have a lot of host plant species. For instance, *Agraulis vanilla* has 50 reported host plants and *Heliconius numata* 30 (Kozak, 2016). We conducted our analysis at the subspecies level because locally subspecies actually use much less host plants. In our study, generalist is subspecies that feed on more than 5 host plant species whereas specialist subspecies feed on 5 or less host plant species. We adjusted this classification based on the literature.

## 3 RESULTS

### 3.1 Large variations in the concentration of neo‐synthesized and sequestered CGs in wild Heliconiini

Across the 375 analyzed Heliconiini samples, nine CGs were identified and important variation in the CG profile was detected between genera and species (Table 2). Important variation of CG profile was also detected within species, notably among different subspecies (MANOVA, ${\text{Pillai}}_{303}^{49}$ = 3.513, *p* < .001).

**Table 2:** Mean concentration and associated standard deviation for each compound detected

Species	Linamarin	Lotaustralin	Epilotaustralin	Tetraphyllin B	Epivolkenin	Tetraphyllin A	Tetraphyllin A stereoisomer	Gynocardin	Dihydrogynocardin
*Agraulis vanillae*	17.91 ± 8.43	5.10 ± 8.80	3.74 ± 7.58	0.34 ± 0.84	0.00	0.00	0.00	0.00	0.00
*Agraulis vanillae luciana*	17.28 ± 5.17	1.22 ± 0.91	0.24 ± 0.48	0.00	0.00	0.00	0.00	0.00	0.00
*Agraulis vanillae vanillae*	19.16 ± 16.43	12.85 ± 14.28	10.74 ± 11.81	1.02 ± 1.45	0.00	0.00	0.00	0.00	0.00
*Dione juno*	13.28 ± 7.08	2.98 ± 3.62	1.40 ± 1.38	0.00	0.00	0.00	0.00	0.00	0.00
*Dione juno huascuma*	16.65 ± 2.41	3.88 ± 2.37	2.57 ± 3.13	0.00	0.00	0.00	0.00	0.00	0.00
*Dione juno miraculosa*	12.50 ± 7.63	2.77 ± 3.90	1.13 ± 0.59	0.00	0.00	0.00	0.00	0.00	0.00
*Dryadula phaetusa*	7.96 ± 3.32	1.57 ± 1.19	0.00	0.00	0.03 ± 0.07	0.00	0.00	0.00	0.00
*Dryas iulia moderata*	7.47 ± 10.32	2.51 ± 4.58	0.09 ± 0.28	0.63 ± 1.71	7.50 ± 10.56	0.31 ± 1.37	0.00	0.13 ± 0.61	0.00
*Eueides aliphera aliphera*	30.66	6.40	0.00	0.00	0.00	0.00	0.00	0.00	0.00
*Eueides isabella*	54.18 ± 31.07	8.39 ± 5.53	0.30 ± 0.75	0.07 ± 0.36	0.73 ± 3.60	0.00	0.00	0.00	0.00
*Eueides isabella dissoluta*	58.11 ± 33.81	7.93 ± 6.11	0.38 ± 0.83	0.09 ± 0.40	0.93 ± 4.04	0.00	0.00	0.00	0.00
*Eueides isabella eva*	43.38 ± 4.58	10.55 ± 2.30	0.00	0.00	0.00	0.00	0.00	0.00	0.00
*Eueides isabella hippolinus*	33.02 ± 6.55	9.58 ± 0.68	0.00	0.00	0.00	0.00	0.00	0.00	0.00
*Eueides lampeto acacetes*	38.15 ± 1.47	2.20 ± 1.83	0.00	0.00	0.00	0.00	0.00	0.00	0.00
*Eueides lybia lybia*	37.51 ± 8.33	7.15 ± 2.71	0.00	0.00	0.00	0.00	0.00	0.00	0.00
*Eueides tales calathus*	12.45	5.48	0.81	0.00	0.00	0.00	0.00	0.00	0.00
*Heliconius aoede cupidineus*	0.40 ± 1.15	0.11 ± 0.28	0.03 ± 0.12	2.02 ± 9.49	31.04 ± 14.70	0.00	0.00	0.00	0.17 ± 0.39
*Heliconius burneyi jamesi*	9.23	2.98	0.95	0.00	0.00	1.67	0.77	0.00	0.00
*Heliconius charithonia vazquezae*	45.18 ± 13.08	45.78 ± 24.24	4.91 ± 0.45	0.00	0.00	0.00	0.00	0.00	0.00
*Heliconius congener congener*	0.45 ± 0.77	0.55 ± 0.95	0.12 ± 0.20	0.00	25.96 ± 22.48	15.74 ± 26.85	0.00	0.00	0.00
*Heliconius demeter joroni*	3.93 ± 1.94	2.08 ± 0.16	0.00	1.08 ± 1.52	30.45 ± 3.79	0.48 ± 0.68	0.00	0.00	0.00
*Heliconius doris*	25.44 ± 7.88	7.73 ± 8.58	0.11 ± 0.25	0.00	0.00	0.00	0.00	0.00	0.00
*Heliconius doris doris*	24.37 ± 8.28	5.50 ± 7.36	0.09 ± 0.27	0.00	0.00	0.00	0.00	0.00	0.00
*Heliconius doris viridis*	27.56 ± 7.64	12.20 ± 10.18	0.13 ± 0.27	0.00	0.00	0.00	0.00	0.00	0.00
*Heliconius eleuchia primularis*	6.84 ± 9.67	3.07 ± 4.35	1.14 ± 1.61	0.00	0.00	12.34 ± 6.92	4.34 ± 1.86	0.00	0.00
*Heliconius erato*	3.77 ± 10.68	3.58 ± 11.12	0.39 ± 1.49	1.58 ± 3.91	6.07 ± 10.02	0.58 ± 2.35	0.00	0.00	0.03 ± 0.21
*Heliconius erato cyrbia*	15.42	12.89	4.97	0.00	0.00	14.30	0.00	0.00	0.00
*Heliconius erato demophoon*	38.82 ± 9.22	41.21 ± 5.29	3.50 ± 3.94	0.00	0.00	2.11 ± 2.04	0.00	0.00	0.00
*Heliconius erato emma*	2.57 ± 3.57	0.81 ± 1.60	0.00	0.47 ± 0.66	13.49 ± 18.06	0.22 ± 0.50	0.00	0.00	0.00
*Heliconius erato favorinus*	0.19 ± 0.41	0.08 ± 0.45	0.00	1.97 ± 4.38	5.65 ± 8.53	0.05 ± 0.27	0.00	0.00	0.04 ± 0.23
*Heliconius eratosignis ucayalensis*	1.87 ± 1.66	0.79 ± 0.56	0.00	5.51 ± 4.41	30.54 ± 8.74	1.89 ± 2.61	0.00	0.00	0.00
*Heliconius ethilla aerotome*	26.30 ± 10.38	5.02 ± 2.66	0.74 ± 0.73	0.00	0.00	0.00	0.00	0.00	0.00
*Heliconius hecale*	17.88 ± 7.53	13.66 ± 8.23	2.45 ± 2.98	0.23 ± 0.70	0.33 ± 0.99	0.00	0.00	0.00	0.00
*Heliconius hecale felix*	10.94 ± 5.66	5.02 ± 3.93	0.00	0.00	0.00	0.00	0.00	0.00	0.00
*Heliconius hecale melicerta*	19.74 ± 7.71	16.62 ± 8.05	2.79 ± 3.17	0.35 ± 0.86	0.50 ± 1.22	0.00	0.00	0.00	0.00
*Heliconius hecale zuleika*	20.59	13.16	5.29	0.00	0.00	0.00	0.00	0.00	0.00
*Heliconius hewitsoni*	0.00	0.09 ± 0.16	0.00	0.00	28.91 ± 4.55	0.00	0.00	0.00	0.00
*Heliconius himera*	3.44 ± 2.77	1.66 ± 1.95	0.91 ± 1.28	0.77 ± 1.06	1.24 ± 2.77	0.00	0.00	2.22 ± 3.28	0.00
*Heliconius melpomene*	18.51 ± 11.97	9.79 ± 11.69	1.13 ± 1.87	3.31 ± 4.66	0.39 ± 1.87	0.13 ± 0.49	0.00	0.10 ± 0.54	0.38 ± 1.48
*Heliconius melpomene aglaope*	24.60	13.09	1.75	4.57	0.00	1.75	0.00	0.00	0.00
*Heliconius melpomene amaryllis*	15.82 ± 8.52	5.56 ± 3.31	0.67 ± 0.96	3.78 ± 5.10	0.55 ± 2.20	0.10 ± 0.44	0.00	0.00	0.00
*Heliconius melpomene amaryllis aglaope (hybrid)*	10.15 ± 5.99	5.25 ± 2.47	0.71 ± 0.65	4.03 ± 4.27	0.00	0.00	0.00	0.00	0.00
*Heliconius melpomene rosina*	37.34 ± 15.00	34.61 ± 15.06	3.74 ± 3.93	0.00	0.00	0.00	0.00	0.72 ± 1.45	2.74 ± 3.44
*Heliconius numata*	14.52 ± 8.33	5.29 ± 3.75	0.74 ± 1.10	0.15 ± 0.65	2.96 ± 7.20	0.00	0.00	0.00	0.00
*Heliconius numata arcuella*	12.26 ± 4.15	9.16 ± 2.68	3.58 ± 0.73	0.00	0.00	0.00	0.00	0.00	0.00
*Heliconius numata bicoloratus*	14.41 ± 9.00	4.92 ± 3.42	0.58 ± 0.86	0.24 ± 0.88	4.76 ± 9.02	0.00	0.00	0.00	0.00
*Heliconius numata lyrcaeus*	6.72	5.27	2.19	0.00	0.00	0.00	0.00	0.00	0.00
*Heliconius numata tarapotensis*	15.5 ± 8.49	5.23 ± 4.55	0.47 ± 0.81	0.00	1.09 ± 3.79	0.00	0.00	0.00	0.00
*Heliconius numata zobryssi*	17.13	5.27	0.00	0.61	0.00	0.00	0.00	0.00	0.00
*Heliconius pachinus*	17.01 ± 4.95	6.31 ± 1.97	3.98 ± 2.72	0.00	0.00	0.00	0.00	0.00	0.00
*Heliconius pardalinus*	17.80 ± 8.89	5.38 ± 3.18	0.42 ± 0.63	0.00	0.00	0.00	0.00	0.00	0.00
*Heliconius pardalinus butleri*	17.56 ± 0.92	5.84 ± 0.28	0.97 ± 1.37	0.00	0.00	0.00	0.00	0.00	0.00
*Heliconius pardalinus sergestus*	17.83 ± 9.55	5.32 ± 3.41	0.34 ± 0.51	0.00	0.00	0.00	0.00	0.00	0.00
*Heliconius sara*	10.59 ± 10.80	8.07 ± 8.41	1.49 ± 3.00	0.94 ± 6.19	38.17 ± 40.18	1.55 ± 9.95	0.00	0.81 ± 2.71	0.56 ± 1.33
*Heliconius sara magdalena*	12.98 ± 14.65	11.63 ± 8.28	1.88 ± 0.89	0.00	75.92 ± 40.87	0.00	0.00	0.00	1.3 ± 1.8
*Heliconius sara sara*	10.28 ± 10.41	7.61 ± 8.42	1.43 ± 3.17	1.07 ± 6.58	33.20 ± 37.86	1.76 ± 10.58	0.00	0.92 ± 2.87	0.46 ± 1.25
*Heliconius telesiphe sotericus*	9.42 ± 3.59	3.26 ± 2.26	0.57 ± 0.53	0.00	0.00	0.00	0.00	0.00	0.00
*Heliconius timareta*	9.76 ± 1.85	5.76 ± 3.36	1.53 ± 1.82	0.00	0.00	0.00	0.00	0.00	0.00
*Heliconius timareta thelxinoe*	7.82	1.92	0.00	0.00	0.00	0.00	0.00	0.00	0.00
*Heliconius timareta timareta*	10.73 ± 1.11	7.69 ± 0.68	2.30 ± 1.75	0.00	0.00	0.00	0.00	0.00	0.00
*Heliconius wallacei flavescens*	20.09 ± 8.87	6.37 ± 2.44	0.08 ± 0.26	0.03 ± 0.10	0.00	0.00	0.00	0.00	0.00
*Heliconius xanthocles*	11.70 ± 10.41	6.15 ± 6.45	0.00	0.25 ± 0.43	14.80 ± 25.64	0.09 ± 0.16	0.00	1.80 ± 1.60	0.00
*Heliconius xanthocles melior*	0.00	0.00	0.00	0.75	44.41	0.27	0.00	0.00	0.00
*Heliconius xanthocles zamora*	17.55 ± 3.41	9.23 ± 5.12	0.00	0.00	0.00	0.00	0.00	2.70 ± 0.52	0.00
*Philaethria diatonica*	9.85 ± 0.34	1.34 ± 0.79	0.00	0.00	0.00	0.00	0.00	0.00	0.00
*Philaethria dido*	7.87 ± 3.54	2.68 ± 0.93	0.00	0.00	0.00	0.00	0.00	0.00	0.00
*Philaethria dido dido*	5.37	2.03	0.00	0.00	0.00	0.00	0.00	0.00	0.00
*Philaethria dido panamensis*	10.38	3.33	0.00	0.00	0.00	0.00	0.00	0.00	0.00

Note

We present data for both species and subspecies. CG concentrations are given in µg/mg of dried body mass.

**Figure 2:**
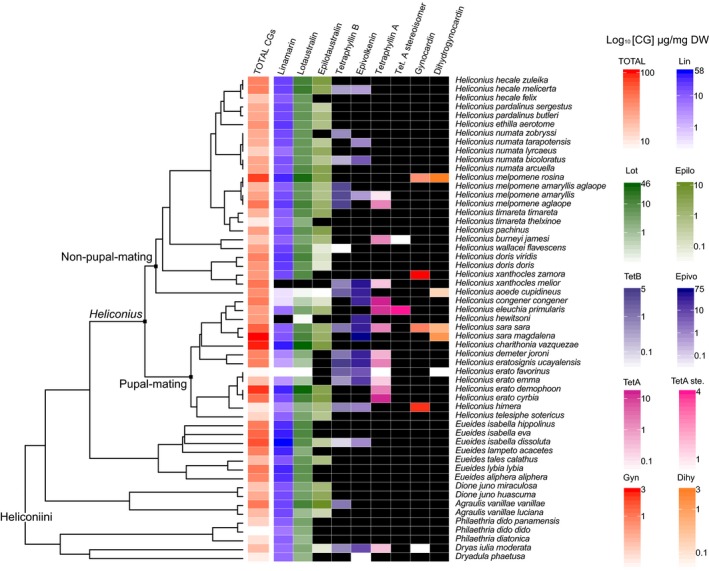
Qualitative and quantitative variations for the nine studied CGs across Heliconiini subspecies. Phylogenetic tree is adapted from (Kozak et al., 2015)**.** The left column represents the total CG mean concentration (*n* = 375 individuals in 55 subspecies). Following column presents the average of each CG concentration. Concentrations are in µg of CG per mg of dried weigh (body + wings) in a logarithmic scale. A black box signifies either the absence of the CG or insufficient data for measurement. A colored filled box indicates that the corresponding CG has been reported in at least one individual of the species. Color gradient is from white corresponding to the minimum reported concentration to the darkest color corresponding to the maximal reported concentration

Regarding putatively synthesized aliphatic CGs, linamarin was detected in all 32 out of 33 species, whereas lotaustralin was in all species (Figure 2). However, the concentration of linamarin was significantly different between species (ANOVA, $F_{342}^{32}$ = 13.77, *p* < .001), and individuals from the genus *Eueides* had statistically significant higher linamarin concentration compared with other genera (ANOVA, $F_{368}^{6}$ = 35.46, *p* < .001; Tukey's HSD, *p* < .001). Similarly, lotaustralin concentrations differed among species (ANOVA, $F_{342}^{32}$ = 4.324, *p* < .001). Another aliphatic CG, epilotaustralin, was detected in *Heliconius*, *Eueides*, *Dione, Agraulis,* and *Dryas* genera, with significant variation in concentration among species (ANOVA, $F_{342}^{32}$ = 2.618, *p* < .001). These three putatively synthesized CGs were found at the highest levels in *H. charithonia,* which also did not contain any putatively sequestered CGs in the two analyzed individuals.

Six putatively sequestered CGs from *Passiflora* host plants were measured: tetraphyllin A, a diastereoisomer of tetraphyllin A, tetraphyllin B, a diastereoisomer of tetraphyllin B called epivolkenin, gynocardin, and dihydrogynocardin. The diastereoisomer of the tetraphyllin A could be deidaclin, because this molecule is also produced by *Passiflora* species used as host plant by Heliconiini butterflies (Jaroszewski et al., 2002; Spencer, Seigler, & Domingo, 1983; Tober & Conn, 1985). We also searched for the aromatic CGs amygdalin as it has been measured in few analyzed *Passiflora* species (Castro, Zagrobelny, et al., 2019; Chassagne, Crouzet, Bayonove, & Baumes, 1996), but we did not find aromatic CGs in Heliconiini butterflies, as previously reported in reared *H. melpomene* (Castro, Demirtas, et al., 2019). The diversity of putatively sequestered CGs and their important variations between species in the wild (MANOVA, ${\text{Pillai}}_{342}^{32}$ = 1.735, *p* < .001) highlight that CG sequestration is widely distributed among the Heliconiini tribe, and may depend on local host plant availability and host plant adaptation.

### 3.2 Evolution of cyanogenic glucoside profiles in Heliconiini

CG profiles in Heliconiini species (*n* = 33) displayed a weak but significant phylogenetic signal (*K*_mult_ **= 0.311, *p* = .023). In *Heliconius*, the largest genus in the Heliconiini radiation, the phylogenetic signal was also moderate but still significant (*K*_mult _= 0.558, *p* = .029). In the genus *Heliconius*, many species have subspecies living in different localities, where individuals display locally mimetic color patterns. To test whether the natural selection act on the evolution of defenses due to the evolution of mimetic color pattern, we then estimated the phylogenetic signal in the genus *Heliconius* at the taxonomic level of subspecies (*n* = 55). We observed that the phylogenetic signal of mean CG concentrations then become weaker and nonsignificant (*K*_mult_ = 0.084, *p* = .055), probably because of important variation among subspecies, consistent with the hypothesis of variations in the strength of selection regarding defenses in different mimicry rings. Intraspecific variations of defenses between localities (four countries, MANOVA, ${\text{Pillai}}_{371}^{3}$ = 0.546, *p* < .001) could then be explained by either (a) variation in the mimetic community abundance and levels of defenses in comimetic species or (b) variation in host plant availability or host plant specialization levels.

### 3.3 Ecological factors influencing the evolution of cyanogenic glucoside profiles in Heliconiini

**Figure 3:**
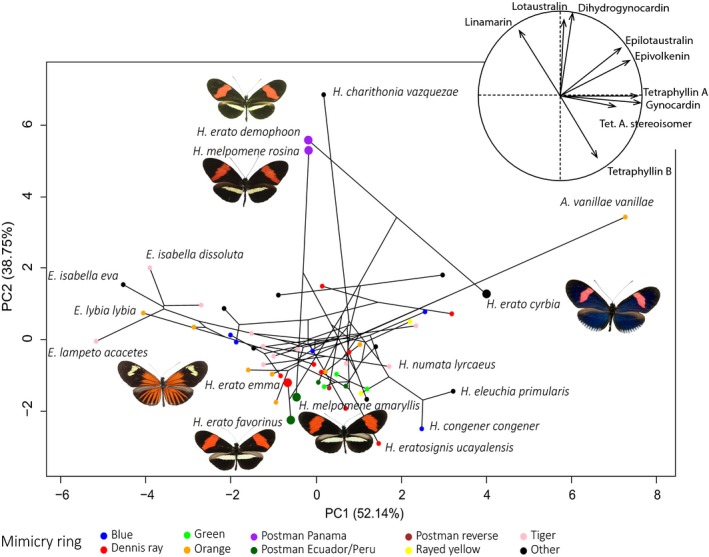
Phylochemospace depicting the relationships between phylogenetic history and the mean CG concentration in Heliconiini subspecies. Visualization in two dimensions of the distribution of the variation in CG profiles. Dark line represents the phylogenetic tree modified from Kozak et al., (2015) to plot subspecies used in our analyses (*n* = 55 subspecies). Dots are mean imputed CG profile per subspecies. Color indicates the mimicry ring subspecies belong to (Table 1). *Heliconius erato* subspecies from distinct mimicry rings also differ in their mean chemical profiles (*H. e. cyrbia* in the “Other” mimicry ring from Ecuador, *H. e. emma* from Dennis‐ray ring from Peru, *H. e. favorinus* from Postman ring from Peru and *H. e. demophoon* from Postman ring from Panama). *H. erato* and *H. melpomene* subspecies have increased size dot and are illustrated by a photo

To explore the contribution of shared ancestry on one hand, and of ecological factors influencing the evolution of defenses on CG variation on the other hand, we drew a phylochemospace displaying average chemical profile of the different subspecies (Figure 3). We observed that subspecies belonging to distinct mimicry rings sometimes had very distinct chemical profiles, *for example H. erato favorinus* (*n* = 31), *H. erato emma* (*n *= 5), *H. erato demophoon* (*n* = 3), and *H. erato cyrbia* (*n* = 1) (MANOVA, ${\text{Pillai}}_{36}^{3}$ = 2.002, *p* < .001). The distantly related comimics *H. melpomene rosina* (*n* = 4) and *H. erato demophoon* (*n* = 3) are located closely on the phylochemospace (Figure 3), because of their similar chemical profiles (MANOVA, ${\text{Pillai}}_{5}^{1}$ = 0**.**615, *p* = .621). Similarly, *H. melpomene amaryllis* (*n* = 21) and its comimic *H. erato favorinus* (*n* = 31) are located closely in the phylochemospace but their CG profiles were still significantly different (MANOVA, ${\text{Pillai}}_{50}^{1}$ = 0.759, *p* < .001).

Overall, the mimicry ring was significantly associated with CG profiles, suggesting that individuals from different species belonging to the same mimicry ring had similar chemical defenses (Table 3). Nevertheless, this association was no longer significant when controlling for shared ancestry, suggesting that the similarity in defense levels could be mainly due to increased phylogenetic proximity within mimicry rings (Table 3).

**Table 3:** Comparisons of CG profile (MANOVA) between and among mimicry rings and host plant specialization levels

MANOVA on mean per subspecies (*n* = 55)	Regular	Phylogenetic
	Mimicry ring^a^	
	${\text{Pillai}}_{36}^{9}$ = 2.736, *p* < .001	${\text{Pillai}}_{36}^{9}$ = 2.736, *p* < .582
	Host plant specialization^a^	
	${\text{Pillai}}_{53}^{1}$ = 0.446, *p* < .001	${\text{Pillai}}_{53}^{1}$ = 0.446, *p* < 1.000
MANOVA on interindividual variation (*n* = 375)	Regular	
	Mimicry ring^a^	
	${\text{Pillai}}_{364}^{10}$ = 1.209, *p* < .001	
	Host plant specialization^a^	
	${\text{Pillai}}_{373}^{1}$ = 0.165, *p* < .001	

Note:

To compare the effect of mimicry rings and host plant specialization on CG profiles with phylogenetic effect, we performed a MANOVA using the mean concentration per subspecies (n = 55 subspecies). Then MANOVA was performed on CG profiles using the whole dataset to test for interindividual variation (n = 375 individuals), without testing the effect of phylogeny.

a Note that each factor was tested using an independent MANOVA.

**Figure 4:**
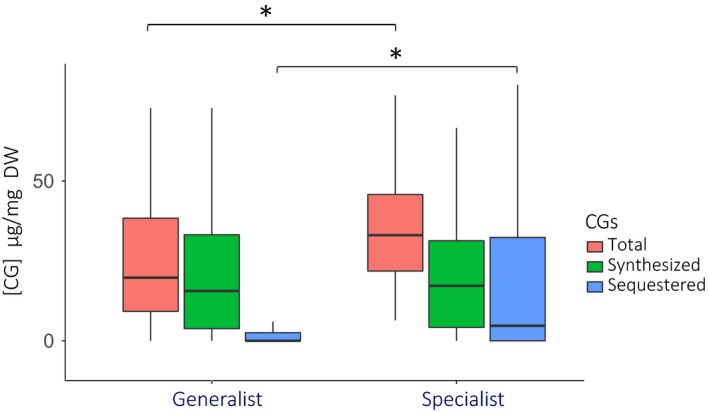
Amount of chemical defenses according to host plant specialization. CG concentrations are given in µg/mg of dried body mass. We pooled generalist subspecies (*n* = 210 individuals distributed in 32 subspecies) on the left and specialist subspecies (*n* = 165 individuals distributed in 23 subspecies) on the right. We represented the total amount of CG (red boxplot) that sums synthesized (green boxplot) and sequestered (blue boxplot) CG concentrations. Asterix shows significant statistical difference

The level of host plant specialization could also influence the evolution of defense in Heliconiini. Indeed, we noticed that the chemical profiles of butterflies depended on their level of host plant specialization, although this effect is mostly driven by phylogenetic proximity (Table 3). Because there is substantial geographical variation in the level of specialization, we also compared chemical defenses among subspecies: individuals from host plant‐specialist subspecies were generally more chemically defended (mean total [CGs] = 39.2 µg/mg DW) than generalist (26.5 µg/mg DW; Table 3, Figure 4). Specialist subspecies sequestered more CGs (19.2 µg/mg DW) than generalist subspecies (3.8 µg/mg DW; ANOVA, $F_{373}^{1}$ = 53.01, *p* < .001). This is pointing at the effect of host plant specialization on chemical profiles that could substantially vary among localities (note that such specialization could depend on the butterfly ability to choose and survive on different plants but also on the local host plant availability).

### 3.4 Geographical variation in chemical profiles

**Figure 5:**
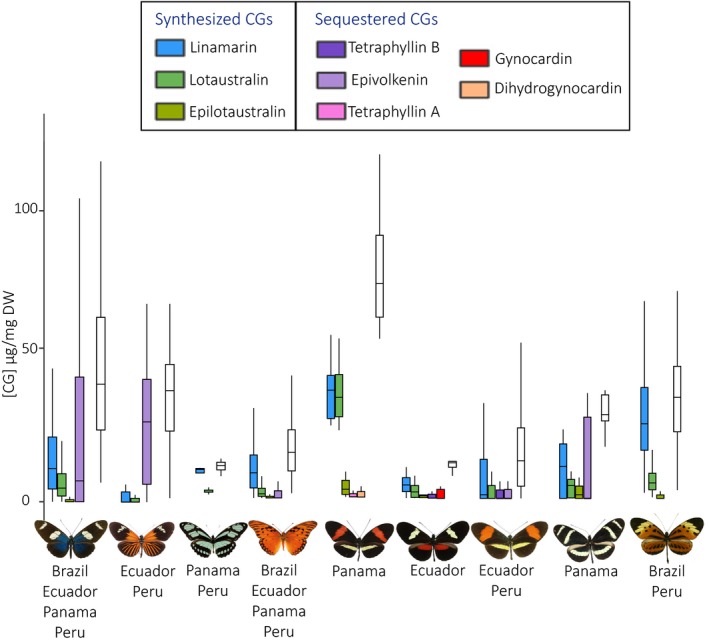
Variation in chemical profiles of individuals from the nine studied mimicry rings, located in different regions of Central and South America. CG concentrations are given in µg/mg DW. Mimicry rings from left to right, with illustrations of the color pattern: blue (6 subspecies, *n* = 66 individuals), Dennis ray (10 subspecies, *n* = 39), green (3 subspecies, *n* = 4), orange (8 subspecies, *n* = 73), postman Panama (2 subspecies, *n* = 7), postman reverse (2 subspecies, *n* = 6), postman from Ecuador and Peru (5 subspecies, *n* = 57), rayed yellow (2 subspecies, *n* = 7), and tiger (11 subspecies, *n* = 78). White boxplots are mean total CG concentration

**Figure 6:**
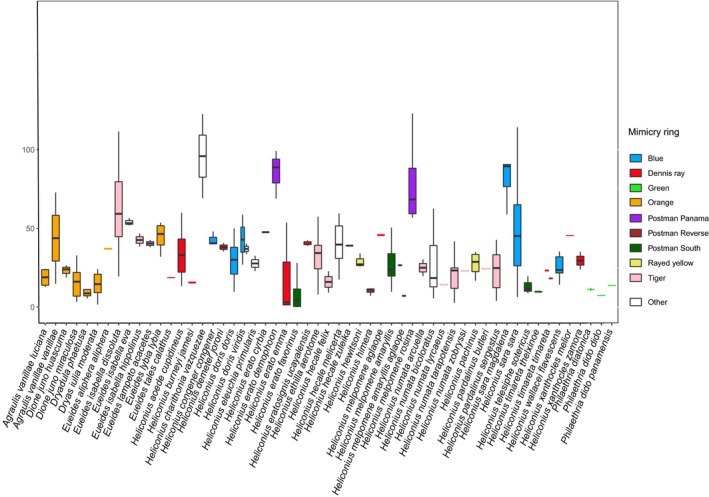
Total CG concentration per subspecies. Concentrations are given in µg/mg DW. Boxplot colors correspond to the associated mimicry ring with legend on the right. Subspecies are listed in alphabetical order from left to right (*n* = 55 subspecies)

In general, variation in CGs was lower within than between mimicry rings (Table 3). Mimicry rings are composed of different species found in sympatry, they can therefore differ in local abundance but also in host plants availability. Mimetic communities exhibiting the same color pattern (*e.g.,* postman color pattern, Figure 5) are composed of similar species, but still display strikingly different chemical profiles (Figures 5 and 6). Both color pattern and locality indeed have a significant association with chemical profiles, as well as the interaction between these two factors, even when controlling for the species effect (Table 4). This suggests that geographical variations in local abundances of mimetic patterns and/or in local host plants availability and specialization levels may influence the defenses of Heliconiini butterflies.

**Table 4:** Variation of CG chemical profile between individuals (*n* = 375)

Regular MANOVA on interindividual variation (*n* = 375)						
	*df*	Pillai	*F* statistic	Degrees of freedom of the numerator	Degrees of freedom of the denominator	*p*‐value associated with the *F* statistic
Colour pattern	9	1.455	$F_{325}^{9}$ = 6.965	81	2,925	.001
Locality	3	1.167	$F_{325}^{3}$ = 22.544	27	957	.001
Colour pattern + Locality	29	0.540	$F_{325}^{8}$ = 2.607	72	2,592	.001
Species	8	2.371	$F_{325}^{28}$ = 4.153	252	2,925	.001
Specialization	1	0.247	$F_{325}^{1}$ = 11.546	9	317	.001

Note

MANOVA tests if there is difference for the CG chemical profiles between groups (listed in left column). Residuals = 325.

## 4 DISCUSSION

### 4.1 Phylogenetic history partly explains the distribution of CGs across Heliconiini species

We observed that mimicry rings had different levels of CG profiles and total concentrations, but these differences are mostly driven by close phylogenetic relatedness among mimetic species. Our results in wild‐caught individuals are thus consistent with the significant phylogenetic signal in CG profile observed in captive‐bred Heliconiini (Castro, Zagrobelny, et al., 2019). Nevertheless, the phylogenetic signal associated with CG profile is stronger when considering species rather than subspecies, suggesting that despite a strong effect of the divergence between clades (ancient node), substantial variation within species is observed in our wild‐caught individuals, probably driven by ecological factors acting on the different mimetic subspecies.

### 4.2 Geographic variation in mimicry rings impacts CG profiles

The important variation in CG profile observed within species is mostly explained by variations between subspecies living in different geographic range. For instance, Panamanian subspecies of *A. vanillae* and *H. erato* were more chemically defended than Southern subspecies of the same two species. Subspecies generally differ in wing color pattern and geographic distribution, pointing at the influence of ecological factors in shaping the variation in CG concentration profile in Heliconiini. Although *Heliconius* species from the pupal‐mating and nonpupal‐mating clades are phylogenetically distant, they can be involved in the same mimicry ring. This is the case for *H. erato demophoon* and *H. melpomene rosina,* which are part of the postman Panama mimicry ring and presented similar CG profiles, suggesting either an effect of the mimetic interactions and/or of the similarity in local host plant chemistry. By sampling wild butterflies from different countries, our study highlights that host plant interaction and geography are important ecological factors shaping variations in chemical defenses within species.

### 4.3 How host plant specialization shapes chemical defenses

Indeed, host plant range and preference vary locally in some species (Smiley, 1978), so that variation in putatively‐sequestrated CGs in butterflies probably reflects host plant availability and use across sampled localities. For example, *H. melpomene* has a wider range of host plant species in its eastern distribution area. In Central America, it feeds on *P. menispermifolia* or *P. oerstedii* depending on the localities but feeds preferentially on *P. platyloba* in Peru, (Billington, Thomas, & Gilbert, 1990; Jiggins, 2016). This emphasizes the plasticity in the host plant range of many Heliconiini species and the importance of local adaptation with *Passiflora* species. Local patterns in host plant use by Heliconiini are likely reflected in their CG profile.

The binary generalist/specialist classification used here is a rough simplification of the host plant specialization spectrum. Nevertheless, we still observed, as expected, that specialist subspecies had higher concentrations of putatively‐sequestrated CGs (Engler & Gilbert, 2007; Jiggins, 2016). However, we did not detect any correlation between the level of host plant specialization and the synthesis/sequestration balance, contrary to previous studies where synthesis and sequestration were shown to be negatively correlated traits, with fluctuant intensity across the phylogeny (Castro, Zagrobelny, et al., 2019; Engler & Gilbert, 2007).

As CGs are *Passiflora* secondary metabolites, their production may vary in space, time and across tissues depending on abiotic and biotic conditions exert on plant. Thus, reported putatively‐sequestrated CGs in our study on wild butterflies are potentially a subset of the CGs contained in locally available *Passiflora* host plants. The evolution of Heliconiini chemical defense profile would thus be shaped by both host plant specialization of the different butterfly species and available *Passiflora* host plants variations across the geographical areas.

### 4.4 Variability of CG profiles within mimicry rings and Müllerian mimicry

Variation in CG concentrations between mimicry rings observed here had already been reported in a study based on colorimetric assays (to investigate total CG concentration per individual regardless of each CG identity) (Arias et al., 2016). This effect of mimicry on the individuals belonging to different co‐occurring mimicry rings is thus not necessarily equally defended, and potentially perceived with different degrees of aversion by predators. Recently, an experiment using domestic chicks shows that beyond a certain CG concentration, birds learned to avoid the prey at a similar speed (Chouteau, Dezeure, Sherratt, Llaurens, & Joron, 2019). Variations in the level of CGs observed within and among mimicry rings might thus not directly translate into variation in learning behavior by predators, so that the evolution of high chemical defense in some Heliconiini would not necessarily be promoted by natural selection exerted by predators in mimetic prey. Furthermore, it is currently unknown whether predator rejection behavior depends on the total concentration of CG or is mostly shaped by the presence of key CGs with a particularly repellent taste. Chemical defenses are also a complex cocktail (Speed, Ruxton, Mappes, & Sherratt, 2012) with components acting through synergetic or antagonist effects.

Predator communities and strength in predation pressure acting on aposematic prey vary in space and time, as demonstrated in the field using artificial poison frogs and caterpillars (Chouteau & Angers, 2011; Mappes, Kokko, Ojala, & Lindström, 2014). Predator sensibility to detect bitterness of CGs and to endure unpleasant taste varies (Li & Zhang, 2014), as well as their tolerance toward cyanide (Cardoso, 2019). Indeed, based on how hungry they are, avian predators may decide to feed on unpalatable butterflies (Chai, 1986; Marshall, 1908). The geographic variation in chemical profile detected here might therefore be influenced by both host plant availability and composition of predator communities. But the strong phylogenetic signal detected on CG profiles, and the high sensitivity of predator to CG suggests that the evolution of elevated levels of chemical defense is not directly related to color pattern evolution.

## 5 CONCLUSIONS

Our study sheds light on the evolution of CGs in Heliconiini butterflies, and highlights the strong effect of evolutionary history in the variation of CG profile observed between species. Variation in CG profiles between mimicry rings seems to be mostly driven by phylogenetic relatedness between mimetic species. Nevertheless, the strong variation observed between individuals belonging to different mimicry rings within species suggests that other ecological factors might be at play. Some species seem to rely on de novo synthesis only, whereas other species mostly perform CG sequestration from *Passiflora* host plants. Many species rely on a combination of these two pathways for CG acquisition, which contributes to substantial variation of chemical profiles both between species and among species. Geographic variation in host plants, but also abundance of mimicry rings could also influence the CG profile: The individual predation risk is indeed lower in abundant mimicry rings as compared with rare ones (Chouteau, Arias, & Joron, 2016), so that selection for higher distastefulness might be higher in localities where a given mimicry ring is at low density. Ecological studies estimating local host plant and predator community variations, as well as local abundances of mimetic communities, would now be required to better understand the selective pressures shaping chemical defense evolution in mimetic species.

## CONFLICT OF INTEREST

None declared.

## AUTHOR CONTRIBUTIONS

V.L, B.N, O.S, and M.E. conceived the study. O.S, K.M.K, and V.L. collected the specimens. S.B welcomed O.S at the Department of Plant and Environmental Sciences, University of Copenhagen, Denmark, so she could performed the chemical analyses with help from E.C.., O.S. performed statistical analyses and wrote the manuscript with contributions from all authors. All authors participated in constructive discussions and approved manuscript final version.

## Data Availability

Raw data file describing each compound and concentration per individual is available on Dryad following the link: https://doi.org/10.5061/dryad.ghx3ffbjt

## APPENDIX 1

Detailed list of sampled butterfly subspecies (*n* = 375 individuals), with number of females (*n* = 119) and males (*n* = 256) as well as provenance country (Brazil, Ecuador, Panama, or Peru)

**Table:**

Genre	Species	Subspecies	Female	Male	Total	Country	Specialization
*Agraulis*	*vanillae*	*luciana*	1	3	4	Peru	Generalist
*Agraulis*	*vanillae*	*vanillae*	1	1	2	Panama	Generalist
*Dione*	*juno*	*huascuma*	1	2	3	Panama	Generalist
*Dione*	*juno*	*miraculosa*	5	8	13	Peru	Generalist
*Dryadula*	*phaetusa*	NA	2	6	8	Peru/Ecuador	Generalist
*Dryas*	*iulia*	*moderata*	14	24	38	Peru/Panama/Brazil	Generalist
*Eueides*	*isabella*	*dissoluta*	8	11	19	Peru	Generalist
*Eueides*	*isabella*	*eva*	0	3	3	Panama	Generalist
*Eueides*	*isabella*	*hippolinus*	0	2	2	Peru	Generalist
*Eueides*	*lampeto*	*acacetes*	1	1	2	Peru	Generalist
*Eueides*	*aliphera*	*aliphera*	1	0	1	Brazil	Generalist
*Eueides*	*lybia*	*lybia*	0	4	4	Brazil	Generalist
*Eueides*	*tales*	*calathus*	0	1	1	Ecuador	Generalist
*Heliconius*	*aoede*	*cupidineus*	9	13	22	Peru	Specialist
*Heliconius*	*burneyi*	*jamesi*	0	1	1	Peru	Specialist
*Heliconius*	*charithonia*	*vazquezae*	0	2	2	Panama	Generalist
*Heliconius*	*congener*	*congener*	0	3	3	Ecuador	Specialist
*Heliconius*	*demeter*	*joroni*	2	0	2	Peru	Specialist
*Heliconius*	*doris*	*doris*	3	5	8	Peru	Specialist
*Heliconius*	*doris*	*viridis*	2	2	4	Panama	Specialist
*Heliconius*	*eleuchia*	*primularis*	0	2	2	Ecuador	Specialist
*Heliconius*	*erato*	*cyrbia*	0	1	1	Ecuador	Generalist
*Heliconius*	*erato*	*demophoon*	2	1	3	Panama	Generalist
*Heliconius*	*erato*	*emma*	1	4	5	Peru	Generalist
*Heliconius*	*erato*	*favorinus*	11	20	31	Peru	Generalist
*Heliconius*	*eratosignis*	*ucayalensis*	0	3	3	Peru	Specialist
*Heliconius*	*ethilla*	*aerotome*	5	16	21	Peru	Specialist
*Heliconius*	*hecale*	*felix*	0	2	2	Peru	Generalist
*Heliconius*	*hecale*	*melicerta*	2	4	6	Panama	Generalist
*Heliconius*	*hecale*	*zuleika*	0	1	1	Panama	Generalist
*Heliconius*	*hewitsoni*	NA	0	3	3	Panama	Specialist
*Heliconius*	*himera*	NA	2	3	5	Ecuador	Specialist
*Heliconius*	*melpomene*	*aglaope*	1	0	1	Peru	Specialist
*Heliconius*	*melpomene*	*amaryllis*	5	16	21	Peru	Specialist
*Heliconius*	*melpomene*	*amaryllis*aglaope*	1	2	3	Peru	Specialist
*Heliconius*	*melpomene*	*rosina*	1	3	4	Panama	Specialist
*Heliconius*	*numata*	*arcuella*	2	0	2	Peru	Generalist
*Heliconius*	*numata*	*bicoloratus*	4	15	19	Peru	Generalist
*Heliconius*	*numata*	*lyrcaeus*	1	0	1	Peru	Generalist
*Heliconius*	*numata*	*tarapotensis*	2	10	12	Peru	Generalist
*Heliconius*	*numata*	*zobryssi*	0	1	1	Brazil	Generalist
*Heliconius*	*pachinus*	NA	2	2	4	Panama	Generalist
*Heliconius*	*pardalinus*	*butleri*	1	1	2	Peru	Generalist
*Heliconius*	*pardalinus*	*sergestus*	3	11	14	Peru	Generalist
*Heliconius*	*sara*	*magdalena*	2	3	5	Panama	Specialist
*Heliconius*	*sara*	*sara*	16	22	38	Peru/Ecuador/Brazil	Specialist
*Heliconius*	*telesiphe*	*sotericus*	0	3	3	Ecuador	Specialist
*Heliconius*	*timareta*	*thelxinoe*	0	1	1	Peru	Specialist
*Heliconius*	*timareta*	*timareta*	0	2	2	Ecuador	Specialist
*Heliconius*	*wallacei*	*flavescens*	2	8	10	Peru/Brazil	Specialist
*Heliconius*	*xanthocles*	*melior*	0	1	1	Peru	Specialist
*Heliconius*	*xanthocles*	*zamora*	2	0	2	Ecuador	Specialist
*Philaethria*	*diatonica*	NA	0	2	2	Peru	Generalist
*Philaethria*	*dido*	*dido*	0	1	1	Peru	Generalist
*Philaethria*	*dido*	*panamensis*	1	0	1	Panama	Generalist

Some species do not have subspecies name so it was “NA” assigned. Right column “Specialization” indicates whether subspecies are generalists (feed on wide panel of *Passiflora* plants) or specialists (feed on a restricted range of *Passiflora* plants).
